# An attenuation field network for dedicated cone beam breast CT with short scan and offset detector geometry

**DOI:** 10.1038/s41598-023-51077-1

**Published:** 2024-01-03

**Authors:** Zhiyang Fu, Hsin Wu Tseng, Srinivasan Vedantham

**Affiliations:** 1 Department of Medical Imaging, The University of Arizona, 1501 N. Campbell Ave, Tucson, AZ 85724 USA; 2 Department of Biomedical Engineering, The University of Arizona, Tucson, AZ USA

**Keywords:** Computed tomography, Computational science, Translational research, Three-dimensional imaging

## Abstract

The feasibility of full-scan, offset-detector geometry cone-beam CT has been demonstrated for several clinical applications. For full-scan acquisition with offset-detector geometry, data redundancy from complementary views can be exploited during image reconstruction. Envisioning an upright breast CT system, we propose to acquire short-scan data in conjunction with offset-detector geometry. To tackle the resulting incomplete data, we have developed a self-supervised attenuation field network (AFN). AFN leverages the inherent redundancy of cone-beam CT data through coordinate-based representation and known imaging physics. A trained AFN can query attenuation coefficients using their respective coordinates or synthesize projection data including the missing projections. The AFN was evaluated using clinical cone-beam breast CT datasets (n = 50). While conventional analytical and iterative reconstruction methods failed to reconstruct the incomplete data, AFN reconstruction was not statistically different from the reference reconstruction obtained using full-scan, full-detector data in terms of image noise, image contrast, and the full width at half maximum of calcifications. This study indicates the feasibility of a simultaneous short-scan and offset-detector geometry for dedicated breast CT imaging. The proposed AFN technique can potentially be expanded to other cone-beam CT applications.

## Introduction

Cone-beam computed tomography (CT) dedicated to x-ray imaging of the breast is an emerging tool for breast cancer screening and diagnosis^1,2^. Dedicated breast CT (bCT) provides a true tomographic or three-dimensional visualization of the uncompressed breast and thus eliminates the tissue superposition in existing breast imaging modalities including mammography and digital breast tomosynthesis. However, compared to these “gold-standard” imaging tools, bCT suffers from lower in-plane resolution^3,4^. When the mean glandular dose (MGD) is made comparable to that of standard two-view mammography, the increased image noise and the lower spatial resolution reduce the conspicuity of microcalcifications^1^.

**Figure 1:**
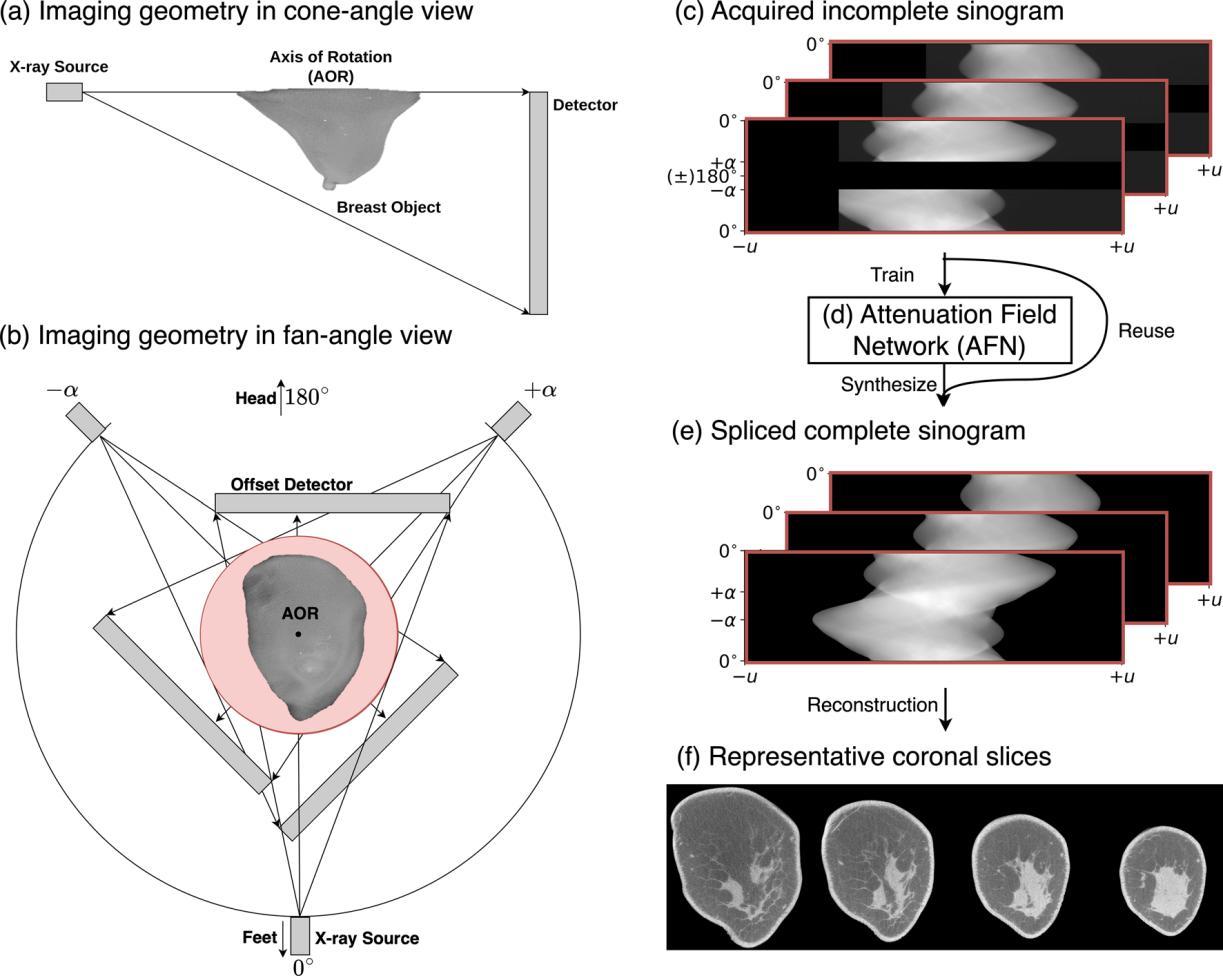
The proposed reconstruction pipeline (**d**–**f**) for a short-scan and offset-detector geometry (a-b) in cone-beam breast CT. (**a**) The half cone-beam geometry in cone-angle view. Patients were imaged in a prone position in our clinical study. (**b**) Fan-angle view. The short scan covers from –$\alpha$ to $+\alpha$ ($\alpha <\pi )$, which is symmetric about the head-feet direction. In addition, data are acquired using an offset detector in each view. (**c**) Such geometry results in acquisitions of incomplete sinogram (stack). (**d**) These incomplete sinogram data are used to train the proposed attenuation field network (AFN) and are reused with the AFN synthesized sinogram to form the spliced complete sinogram in (**e**). Afterwards, we can use any reconstruction methods including analytic methods to obtain a three-dimensional image reconstruction as shown in (**f**).

To improve the system spatial resolution, the latest generation of cone-beam bCT systems use complementary metal–oxide–semiconductor (CMOS) detectors with a smaller pixel pitch (75–150 µm) and exhibit an order of magnitude lower electronic noise than the amorphous silicon flat-panel detectors used in prior generations^5–8^. Nevertheless, the largest CMOS detector has an active area of 30 × 30 cm^2^, which is 10 cm narrower in the fan-angle direction than the 40 × 30 cm^2^ amorphous-silicon flat-panel detector^4^. To accommodate the same imaging field of view (FOV), one may reduce the system magnification by positioning the CMOS detector closer to the axis of rotation (AOR), resulting in a smaller air gap between the breast and the detector, which can increase x-ray scatter and compromise quantitative accuracy. Alternatively, the laterally-shifted (i.e., offset) detector geometry (Fig. 1a,b) can be used to maintain the FOV without modifying the system magnification. In full-scan, the truncated projection data acquired using an offset detector can be compensated using weighting functions owing to the inherent data redundancy of fan-beam data^4,9–13^. The feasibility of an offset detector geometry in full-scan cone-beam bCT using the Feldkamp-Davis-Kress (FDK) algorithm^4^ and a compressed sensing-based iterative reconstruction algorithm^13^ has been demonstrated.

While offset-geometry CMOS detectors can improve spatial resolution and preserve the imaging FOV as well as an optimized air gap, prone-positioning requires a breast CT system with a larger footprint that is difficult to accommodate in typical mammography rooms, and ensuring proper positioning to image the posterior aspects of the breast is time-consuming. In contrast, upright positioning with mammography^14^ or tomosynthesis^3^ has significantly better coverage of the axilla and is easier to position. Thus, an upright bCT that acquires multiple projection views via a short scan, namely, less than 360°, is envisioned. Similar to the truncated projection data acquired with offset detectors, short-scan data can also be compensated using weighting functions, such as Parker weights^15^.

The envisioned system acquires data that are incomplete both on the detector plane and on the trajectory (as shown in Fig. 1b). It is important to note that the compensation weights can only attend to data being incomplete in one dimension. We will show that the use of weighting functions to compensate for either the truncated projection or the short-scan projection fails to reconstruct the incomplete data. Instead, we develop an attenuation field network (AFN) to assist the image reconstruction. AFN adopts the emerging *neural field* paradigm in computer vision, where a scene is represented as a continuous function of coordinates using a multi-layer perceptron (MLP). In the context of CT, neural fields can represent quantities either in the projection domain^16^ or in the image domain^17–20^. Sun et al.^16^ proposed a sinogram field network for sparse-view parallel-beam CT problems. Tancik et al.^17^ briefly demonstrated an indirect supervision approach for the image reconstruction task in two-dimensional CT: a coordinate-based MLP is trained to predict attenuation coefficients, where the network loss is computed between the measured sinogram and the sinogram integrated from the predicted attenuation coefficients. Zang et al.^18^ incorporated this approach as a sinogram prediction prior and demonstrated the framework for parallel-beam CT applications. Both image-domain field networks involve the system forward operator to compute the losses in each iteration. The system operator requires large graphic processing unit (GPU) footprints, especially for high-dimensional problems^21^, e.g., high-resolution cone-beam CT. Recently, Rückert et al.^19^ and Zha et al.^20^ independently proposed image-domain neural field networks for cone-beam CT in which the training is reduced to each ray originating from the x-ray source to a detector pixel. This decomposed training is highly memory efficient and aims to minimize the error between the rendered and the measured projections through a fully differentiable rendering procedure. During inference, a discrete attenuation field is rendered and is regarded as the image reconstruction. We adopt the memory-efficient training yet propose to splice the acquired projections and the network synthesized projections (Fig. 1c,d). The spliced projections are complete and can be used for posterior reconstruction methods of users’ choice (Fig. 1e,f). In addition, hash grid encoding^22^ is included in our network to enhance the learning of high frequency features. We evaluate the technique using 50 clinical breast datasets and demonstrate the feasibility of AFN for cone-beam breast CT with a short-scan and offset-detector geometry.

## Results

### AFN projection synthesis

**Figure 2:**
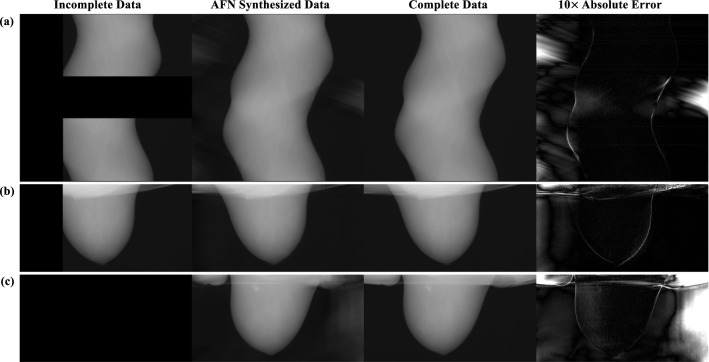
AFN synthesized projection data evaluated in the forms of (**a**) sinogram (data are missing in both the view angle and detector width dimensions), (**b**) acquired projection view demonstrating that the data are incomplete in the detector dimension, and (**c**) unacquired projection view in between 135° to 225°. Projection at 180° is shown here (see Fig. 1b) demonstrating that the data are also incomplete in the view angle dimension. AFN can fully inpaint the sinogram from the incomplete sinogram and only yields high uncertainties outside the anatomical region. In the acquired projection view (**b**), data are partially truncated on the left. AFN outputs visually similar projections as the reference. (**c**) No data are acquired at 180°. In addition, this projection view yields the highest uncertainties since data are most scarce here. Nevertheless, AFN is able to generate a high-fidelity projection view except yielding large errors outside the anatomical region.

A trained AFN can query the respective attenuation coefficient given a volumetric coordinate. The projection data at any detector pixel can be obtained as a line integral along the ray path from the x-ray source to the detector pixel. AFN can thus synthesize projections for an arbitrary imaging geometry including the underlying geometry of the data itself. Figure 2 shows AFN synthesized three-dimensional projections from different view aspects. In a sinogram (Fig. 2a), the short-scan and offset-detector data are incomplete in both the (view) angle and detector width dimensions. AFN inpaints the sinogram with high fidelity and only yields large errors outside the sinogram. Figure 2b illustrates an acquired projection view, where AFN accurately synthesizes 75% of the acquired projections and recovers the missing (25%) projections. Note that, in the error image, no transition artifacts are visible at the truncation except for higher errors towards the chest wall and air on the left. Figure 2c shows that AFN synthesizes a projection view at 180°, where no data are acquired. Note that this unacquired projection view is located at the center of the unacquired region (referring to Fig. 1b), which implies that the data are most scarce here. Therefore, AFN is expected to yield the highest uncertainties in this projection view. When the synthesis errors (near chest wall in Fig. 2b and c) or uncertainties (air area in Fig. 2) are located outside of FOV, the corresponding image reconstruction is free of artifacts within FOV.

### Impact of AFN inpainting

**Figure 3:**
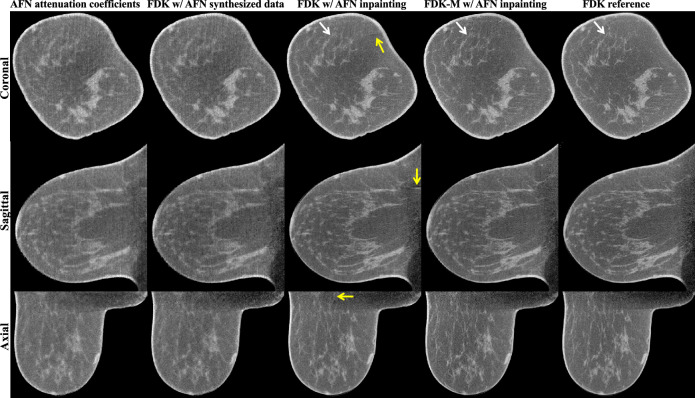
Breast CT image reconstructions (11.8 cm chest-wall diameter) with AFN and/or FDK using incomplete data (columns 1–4) are compared to the FDK reference using complete data (column 5). A trained AFN can either directly output the image volume (column 1) or synthesize projection data for subsequent reconstructions (columns 2–4). When FDK uses the AFN synthesized complete data (column 2), it produces images visually similar to the “AFN attenuation coefficients”, showing a loss of resolution. When the acquired projection data are reused and spliced with AFN synthesized data (column 3), a subsequent FDK reconstruction recovers the lost resolution yet exhibits truncation-like artifacts (yellow arrows) due to the slight inconsistency between AFN synthesized projections and the acquired projections. We incorporated an offset-detector weight into the FDK algorithm, denoted as FDK-M. This weighted FDK reconstruction using AFN inpainted data (column 4) effectively eliminates the line artifacts. The display window is [0.15, 0.35] cm^−1^.

Figure 3 compares the utilization of AFN in the image domain or the projection domain for a representative breast image reconstruction using incomplete data. Column 1 shows the three-dimensional AFN attenuation coefficient map, which was directly queried using the canonical volumetric coordinate grid. Column 2 shows the posterior FDK reconstruction using AFN synthesized data. Both reconstructions illustrate a loss of resolution as well as residual streaks and are of similar visual quality, which in turn validates the efficacy of AFN in learning attenuation representations. In column 3, the acquired incomplete projection data were reused and spliced with AFN synthesized projections to form complete data, namely a process of AFN inpainting. A subsequent FDK reconstruction greatly improved the image resolution yet created truncation-like artifacts in all three planes as indicated by the yellow arrows. We note that these artifacts were probably due to the non-smooth transition between AFN synthesized projections and the acquired projections since no such artifacts were observed in the images of columns 1 and 2. We thus incorporated the offset-detector weighting function proposed by Maaß et al.^11^ into the FDK reconstruction, denoted as FDK-M. As shown in column 4, the inclusion of offset-detector weights eliminates the truncation artifacts in all three planes and further enhances fine structures in the coronal plane (white arrows), compared to the FDK reference reconstructed from complete data in column 5.

### Comparison to FDK with weighting functions

**Figure 4:**
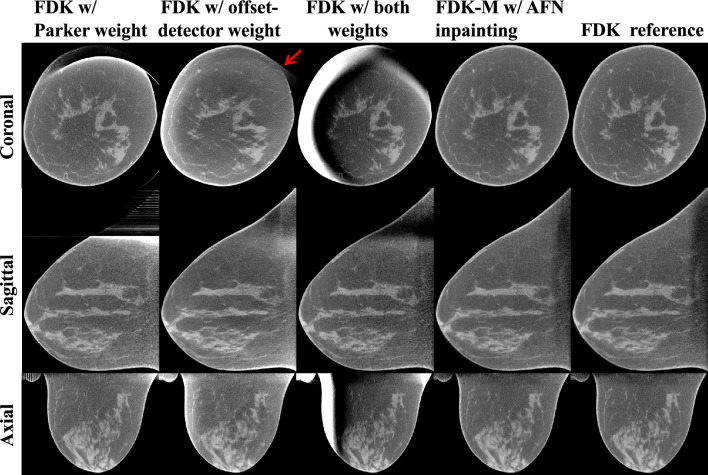
Image reconstructions of a median-size breast (14.5 cm chest wall diameter). Incomplete projection data were used in the four competing methods (columns 1–4) including FDK with Parker weight, FDK with offset-detector weight (denoted as FDK-M), FDK with both weights, and FDK-M with AFN inpainted data. Complete projection data were reconstructed using FDK to obtain the reference (last column). FDK with weighting functions can account for either the short-scan or the offset-detector geometry, leading to residual artifacts and/or inhomogeneous attenuation coefficients as expected. The red arrow indicates the elongated structures due to short scan. FDK with two weights exacerbates the artifacts or inhomogeneities. Lastly, FDK-M with AFN inpainting delivers visually similar images as the reference. The display window is [0.15, 0.35] cm^−1^.

Figure 4 shows the image reconstructions of a medium-sized breast (14.5 cm chest wall diameter). The images obtained using FDK with (modified) Parker weight^23^ exhibit truncation artifacts due to the offset-detector geometry. The images reconstructed using FDK with the offset-detector weight, i.e., FDK-M, manifest inhomogeneous intensities locally and globally due to the short scan. The red arrow indicates the artifacts appearing as elongated structures, which are most profound in the coronal plane. FDK with both weights applied resolves neither the artifacts nor inhomogeneities since either of the two weight is wrongly modulated by the other weight. All weighted FDK reconstructions show noise amplifications, especially near the chest wall, due to the reduced amount of data. In contrast, our FDK-M reconstructions using AFN inpainted projection data are free of the truncation artifacts or the inhomogeneous attenuations and appear less noisy than the two weighted FDK reconstructions, owing to the addition of AFN synthesized projection data.

### Comparison to compressed sensing methods

**Figure 5:**
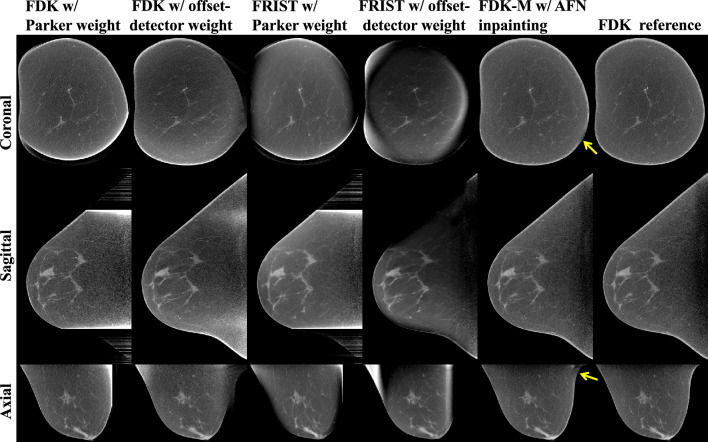
Image reconstructions of a large-size breast (18.3 cm chest wall diameter). Incomplete projection data were reconstructed using FDK with Parker weight, FDK with offset-detector weight (denoted as FDK-M), FRIST initialized using FDK with Parker weight, and FRIST initialized using FDK-M. The incomplete data were inpainted by AFN and further reconstructed by FDK-M. Complete projection data were reconstructed using FDK to obtain the reference (last column). The two FRIST methods further exacerbate the artifacts and/or inhomogeneities despite denoising the breast images. In our proposed method, the breast skin near the chest wall is not fully reconstructed (as indicated by the yellow arrows) since this region is the most under-scanned for the emulated short-scan and offset-detector geometry. The display window is [0.15, 0.35] cm^−1^.

Figure 5 shows the image reconstructions of a large-size breast (18.3 cm chest wall diameter). The compressed sensing method, Fast, total variation-Regularized, Iterative, Statistical Technique (FRIST^24^), suppresses the image noise in the central glandular tissue region. However, FRIST, using either of the two weighted FDK reconstructions as an initialization, further exacerbates the artifacts or inhomogeneities appearing in the weighted FDK reconstructions (columns 1–2) as a result of severe data inconsistency and inefficacy of total variation regularization on image artifacts. In this large-size breast, one side of the breast skin near the chest wall is not fully reconstructed using our proposed method, as indicated by the yellow arrows in the coronal and axial images. It is worth noting that this side of the breast in the axial view is the most under-scanned for this simulated short-scan and offset-detector geometry, and the reconstruction of this region is more vulnerable to artifacts.

### Comparison to fully supervised learning methods

**Figure 6:**
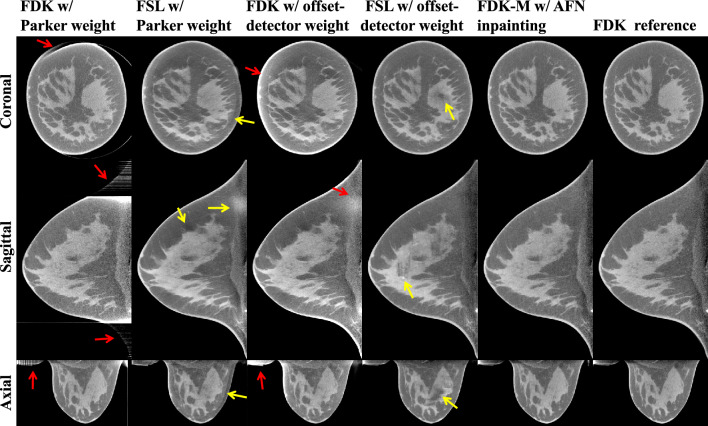
Image reconstructions of a median-size breast (14.5 cm chest wall diameter). Incomplete projection data were reconstructed using FDK with Parker weight, FDK with offset-detector weight (denoted as FDK-M), and two fully supervised learning (FSL) methods, whose network inputs were either of the two weighted FDK reconstructions. The incomplete data were inpainted by AFN and further reconstructed by FDK-M. Complete projection data were reconstructed using FDK to obtain the reference (last column). Using the images reconstructed by FDK w/ Parker weight (column 1), FSL (column 2) alleviates the truncation artifacts indicated by the red arrows in column 1 yet creates additional artifacts as indicated by the yellow arrows in column 2. Similarly, using the images reconstructed by FDK w/ offset-detector weight (column 3), FSL (column 4) addresses the non-homogeneity indicated by the red arrows in column 3 yet generates severe artifacts as indicated by the yellow arrows in column 4. In contrast, reconstructions using AFN inpainted data (column 5) are visually similar to the reference in column 6. The display window is [0.15, 0.35] cm^−1^.

Figure 6 shows the image reconstructions of a medium-size breast (14.5 cm chest wall diameter). Using the images reconstructed by FDK w/Parker weight (column 1), the fully supervised learning (FSL) method (column 2) alleviates the truncation artifacts indicated by the red arrows in column 1 yet creates additional artifacts as indicated by the yellow arrows in column 2. Similarly, using the images reconstructed by FDK w/offset-detector weight (column 3), FSL (column 4) addresses the non-homogeneities indicated by the red arrows in column 3 yet generates severe artifacts as indicated by the yellow arrows in column 4. Notably, the “calcification-like” artifact in the sagittal plane can be detrimental to breast cancer diagnosis. In contrast, reconstructions using AFN inpainted data (column 5) are visually similar to the FDK reference (column 6).

### Quantitative performance compared to the reference FDK method

Table 1 compares the proposed FDK-M reconstruction with AFN inpainting and the reference FDK method using four metrics including the noise variance, the signal difference to noise ratio (SDNR), and the full width at half maximum (FWHM) of calcifications along the mediolateral (ML) direction and the superior-inferior (SI) direction. All four metrics except the SDNR metric ($P=0.194$, Shapiro–Wilk’s test) did not satisfy the normality assumption. There was no significant difference between our proposed method and the reference FDK method for the SDNR metric ($P=0.886$, paired t-test). We performed the non-parametric Wilcoxon-signed rank test for the other three metrics and failed to reject the null hypothesis that the median difference between the two methods is zero due to the p-values being above the significance level of 0.05. All four metrics suggest that our proposed method using incomplete data yields comparable image noise, image contrast, and spatial resolution of calcifications as the FDK reference using complete data.

**Table 1:** Statistical analysis between the proposed AFN method using incomplete data and the reference FDK method using complete data.

	FDK-M w/AFN inpainting	FDK reference	Shapiro–Wilk test	Wilcoxon-signed rank test	Paired t-test
Noise variance (× 10^–5^ cm^−2^)	7*.*55 ± 0*.*31	7*.*69 ± 0*.*35	*P* < 0*.*0001	*P* = 0*.*484	–
SDNR	6*.*36 ± 1*.*65	6*.*35 ± 1*.*83	*P* = 0*.*194	–	*P* = 0*.*886
FWHM-ML (mm)	1*.*68 ± 0*.*72	1*.*61 ± 0*.*75	*P* < 0*.*0001	*P* = 0*.*350	–
FWHM-SI (mm)	1*.*55 ± 0*.*64	1*.*69 ± 0*.*63	*P* < 0*.*0001	*P* = 0*.*135	–

Our proposed reconstruction pipeline consists of AFN inpainting and a subsequent FDK with the offset-detector weight (denoted as FDK-M). Fifty breast cases were evaluated, among which 26 cases contain calcifications. The noise variance was estimated in the adipose region. The signal difference to noise ratio (SDNR) was calculated between the adipose and fibroglandular tissues. The full width at half maximum (FWHM) of the calcification was computed along two orthogonal directions (ML: mediolateral; SI: superior-inferior).

### AFN data sufficiency

**Figure 7:**
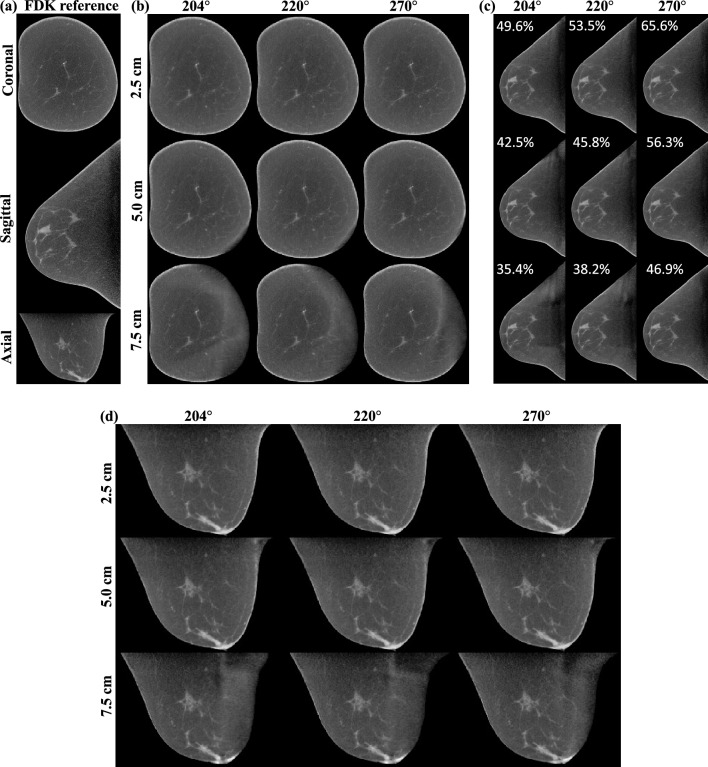
(**a**) the FDK reference in three planes of the same large-size breast shown in Fig. 5. (**b**–**d**) the AFN assisted image reconstructions at different undersampling rates in the coronal (**b**), sagittal (**c**), and axial (**d**) planes, respectively. The undersampling rates in percentage are reported in (**c**) for each combination of detector offset (vertical) and angular coverage (horizontal). AFN reconstructions with a 7.5 cm detector offset (37.5% truncation of a 40 cm detector) yield prominent artifacts in the coronal and axial planes even for the 270° angular coverage. AFN reconstructions with a 5 cm detector offset (25% detector truncation) yield not fully reconstructed breast skin structures near the chest wall, which are less pronounced as the view angle increases. Lastly, AFN reconstructions with a 2.5 cm detector offset (12.5% detector truncation) yield no visible artifacts even for the minimum view angle 204°. The display window is [0.15, 0.35] cm^−1^.

Figure 7a illustrates the FDK reference in three planes of the same large-size breast shown in Fig. 5. Figure 7b–d show the AFN-assisted image reconstructions at different undersampling rates in the coronal (b), sagittal (c), and axial (d) planes, respectively. The undersampling rates in percentage are reported in Fig. 7c for each combination of detector offset (vertical) and angular coverage (horizontal). In each panel of (b)–(d), the image on the top-right corner is associated with the highest rate (65.6%) whereas the image on the bottom-left corner is associated with the lowest rate (35.4%). AFN reconstructions with a 7.5 cm detector offset (37.5% truncation of a 40 cm detector) yield prominent artifacts in the coronal and axial planes even for the 270° angular coverage. AFN reconstructions with a 5 cm detector offset yield not fully reconstruction breast skin structures near the chest wall, which are less pronounced as the view angle increases. Lastly, AFN reconstructions with a 2.5 cm detector offset (requiring a 35 cm wide detector) yield no visible artifacts even for the minimum view angle 204°. This can be promising since the combination of 2.5 cm offset and 204° angular coverage has a lower sampling rate than the current combination of 5 cm offset and 270° angular coverage and will allow for more operation room for the envisioned upright CT system. This data sufficiency analysis indicates that the current cone-beam geometry using AFN-assisted reconstruction is mostly constrained by the detector width.

## Discussion

We have presented a self-supervised image reconstruction technique, AFN, for cone-beam bCT data using a simultaneous short-scan and offset-detector geometry. The resulting incomplete data posed challenges for FDK and compressed sensing methods. Conventional analytical reconstruction methods exploit the projection data redundancy in the view angle and detector width dimensions (as represented in Eq. 3), which requires at least one of the two dimensions to be complete^9–12,24^. In contrast, AFN can exploit the inherent redundancy of projection data through coordinate-based representation (aligned with the physical imaging coordinates), known imaging physics (the Beer-Lambert law based training loss), and high-resolution representation ability (from the hash grid encoding). A trained AFN serves as a continuous attenuation coefficient field and thus can be used to generate novel projection data by integrating all the attenuation coefficients along a ray path. In this study, we demonstrated that AFN can inpaint the short-scan and offset-detector projection data to yield complete data, which can be further reconstructed using the FDK algorithm or its variants with weighting functions. The resulting reconstruction yielded comparable image quality as the reference FDK in terms of image noise, image contrast, and calcification resolution. It is worth noting that the synthesis of AFN is fundamentally different from that of generative adversarial networks (GANs^25^), whose predictions may suffer from hallucinations especially for unseen data. While GANs can synthesize arbitrary projection images from random noise, our AFN intends to infer the underlying attenuation coefficients of a breast by leveraging the inherent correlation or redundancy of the acquired projections of that breast. We underscore that AFN is self-supervised and is independently trained for each breast case, i.e., without the need for a training dataset or data labeling. This also eliminates the generalization problem of supervised networks. For instance, a fully-supervised network trained using sparse-view breast CT data showed reduced performance on calcifications due to being a minority class in the training data^26^. In contrast, we showed unimpaired calcification resolution in our AFN-assisted reconstruction. Moreover, training an AFN is memory efficient and versatile since a minimal training example of AFN is a ray propagation from the x-ray source to a detector pixel. AFN can be suited to learn incomplete CT in other forms, such as low-resolution projection data. The work can potentially be expanded to other cone-beam CT applications with different imaging geometries.

This work has limitations. First, this is a retrospective study where the data were acquired using an amorphous silicon-based detector as a surrogate for the CMOS detector that we envision using in an upright breast CT system. The lower system noise and the finer pixel size of the CMOS detector may affect the data characteristics. Hence, it needs further evaluation with prospective data acquired using a CMOS detector. Second, x-ray scattering and x-ray beam hardening effects that occur during data acquisition were ignored, and the AFN in this work only considered the primary signal in the network loss function. Future studies could incorporate x-ray scattering or x-ray beam hardening models into the network training to obtain artifact-corrected or -reduced image reconstruction. Third, cone-beam data intrinsically yield less data redundancy farther from the center of the FOV. While the coordinate-based representation of AFN is a powerful tool, AFN reconstruction is more susceptible to artifacts towards the periphery of the FOV, as was observed for large-sized breast images. These artifacts could be potentially suppressed with additional constraints/regularizations (e.g., smooth constraint or low-rank constraint^27^) imposed during training, or with more advanced posterior reconstruction methods than the FDK algorithm, which is left for future work. Finally, unlike supervised deep learning methods, the reconstruction time of our self-supervised AFN consists of training (2 h) and testing (25 min) times, which both have a complexity of $\mathcal{O}(kl)$, where $k$ denotes the number of samples per ray and $l$ denotes the number of rays. We used a relatively large $k$ (= 512) to ensure the network can learn a high-resolution attenuation field. Without compromising the reconstruction performance, the number of samples per ray may be reduced by using exponential stepping^22^ (as opposed to the uniform sampling we used) or by skipping ray-marching in empty (air) spaces^28^.

In conclusion, we have presented a novel self-supervised technique, AFN, for dedicated cone-beam bCT with short-scan and offset-detector geometry. This geometry is aimed for upright breast CT systems employing high-resolution, low-noise detectors. The resulting incomplete data cannot be reconstructed using conventional analytical and compressed sensing methods, whereas our proposed AFN technique yields comparable image quality as the reference obtained using complete data.

## Methods

### Cone-beam CT projection

Suppose the attenuation coefficients of an object to be imaged are denoted by ${\varvec{\mu}}\left({\varvec{r}}\right)\in {\mathbb{R}}^{{N}_{x}\times {N}_{y}\times {N}_{z}}$, where ${\varvec{r}}=(x,y,z)\in {\mathbb{R}}^{3}$ is a three-dimensional coordinate. In a circular cone-beam CT system, the x-ray source rotates on the $xy$ plane with its trajectory denoted by ${\varvec{s}}(\beta )=({D}_{so}{\text{cos}}\left(\beta \right),{D}_{so}{\text{sin}}\left(\beta \right),0)$, where ${D}_{so}$ denotes the distance from the source to the rotation origin, and $\beta \in [\mathrm{0,2}\pi )$ denotes the view angle. Suppose a pixel indexed by $(u,v)$ on the flat-plane detector, its respective coordinate ${\varvec{d}}\left(\beta ,u,v\right)\in {\mathbb{R}}^{3}$ is dependent on the view angle $\beta$ as well. The measured intensity profile $I$, according to the Beer-Lambert’s law, is given by

1 $$I={I}_{0}{e}^{-{\int }_{\overrightarrow{{\varvec{s}}{\varvec{d}}}}{\varvec{\mu}}\left(t\right){\varvec{d}}t},$$

where ${I}_{0}$ is the incident photon count, and $t\in [\mathrm{0,1}]$ is the variable of the integration along the line $\overrightarrow{{\varvec{s}}{\varvec{d}}}$. The intensity profile is usually converted to the *projection* of the object ${\varvec{\mu}}(\cdot )$, that is

2 $${\varvec{p}}\left({\varvec{s}},{\varvec{d}}\right)=-{\text{ln}}\frac{I}{{I}_{0}}={\int }_{0}^{1 }{\varvec{\mu}}\left({\varvec{s}}+t\left({\varvec{d}}-{\varvec{s}}\right)\right)dt.$$

Here only the primary beams are taken into account, and this equation serves as a good approximation when x-ray scattering and x-ray beam hardening are negligible.

### FDK reconstruction with weighting functions

Complete cone-beam projection data are commonly reconstructed using the FDK algorithm for its efficiency in practice. FDK can also be suited for incomplete cone-beam data with proper weighting functions. These weighting functions leverage the inherent redundancy of fan beam data, that is,

3 $${\varvec{f}}\left(\beta ,\gamma \right)={\varvec{f}}\left(\beta +\pi +2\gamma ,-\gamma \right),$$

where ${\varvec{f}}$ denotes the two-dimensional fan data parameterized by view angle $\beta$ and $\gamma$, the angle of the ray relative to the center ray. Weighting functions $w(\cdot )$ are designed to yield boundary continuity and unit total weight between two complementary rays^15^, that is,

4 $$w\left(\beta ,\gamma \right)+w\left(\beta +\pi +2\gamma ,-\gamma \right)=1.$$

Mathematically, Eq. (3) only holds true for the cone-beam data acquired on the central plane. However, two rays are considered complementary to each other if they intersect at the central plane and stay within a plane that is perpendicular to the central plane^29^. The same $w(\cdot$) in Eq. (4) can be applied to each detector row of cone-beam data independently.

The weight function can be incorporated into the FDK algorithm before or after the convolution (i.e., filtering) step, denoted as pre-convolution or post-convolution method, respectively^9^. Post-convolution method is usually combined with a proceeding step that fills up all the missing data such that the convolution step produces no extra artifacts. In this study, we used a modified Parker weight^23^ for short-scan data and an offset-detector weight^11^ for truncated data. The domain of the weighting function is $[-{\gamma }_{\text{max}},{\gamma }_{\text{max}}]$, where ${\gamma }_{\text{max}}$ represents the half-fan angle. The first half of the function ${w}_{L}(\cdot )$ defined on $[{-\gamma }_{\text{max}}, 0]$ yields an S-shape:

5 $${w}_{L}\left(\gamma \right)=\frac{1}{4}\left\{\begin{array}{ll}0 & \quad {\text{if}} \gamma \le {-\gamma }_{t}\\ 1+s(2\frac{\gamma -{\gamma }_{t}}{{\gamma }_{s}-{\gamma }_{t}}-1)& \quad\text{else if }\gamma \le {-\gamma }_{s}\\ 2& \quad\text{else }\gamma \le 0\end{array}\right.,$$

where $s\left(x\right)={\text{sin}}(\frac{1}{2}\pi x)$, ${\gamma }_{t}$ denotes the truncation position, and ${\gamma }_{s}$($<{\gamma }_{t}$) is a hyperparameter that controls the smooth transition region $\left[-{\gamma }_{t},-{\gamma }_{s}\right]$ provided by the weighting function. The second half, defined in $[0, {\gamma }_{\text{max}}]$, is a duplicate of ${w}_{L}$ with reflection and a constant offset, that is, ${w}_{R}(\gamma )=2-{w}_{L}(-\gamma )$. The offset-detector weight along with the modified Parker weight both contain a central plateau for improved noise reduction when the angular coverage is greater than $180^\circ +2{\gamma }_{\text{max}}$^23^.

### Attenuation field network (AFN) training, reconstruction, and synthesis

**Figure 8:**
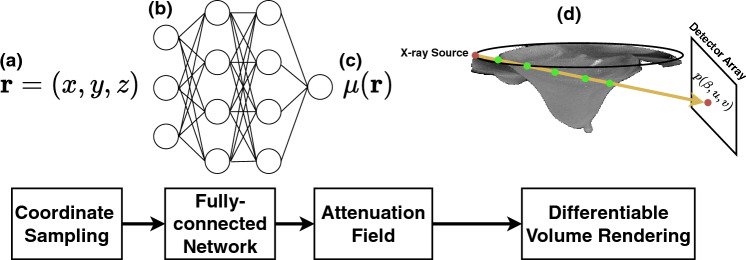
An overview of our attenuation field network (AFN). We map a coordinate vector (**a**) through a fully connected network (**b**) to the respective attenuation coefficient (**c**). The training procedure of AFN coincides with the conventional ray-tracing algorithm. We sample points along a ray path to render the projection intensity at the ray end according to the Beer-Lambert’s law. This rendering procedure is fully differentiable, allowing us to optimize our attenuation representations by minimizing the error between the synthesized projection and the acquired projection. The training is done until we iterate the rendering and optimization steps over all the acquired data.

Our attenuation field network (AFN) is designed to represent the attenuation coefficient ${\varvec{\mu}}({\varvec{r}})$ using its respective physical coordinate ${\varvec{r}}$ through a shallow fully connected network as shown in Fig. 8. The training procedure of AFN strictly follows the cone-beam projection acquisition procedure described in Eq. (2). A minimum training sample of AFN is a ray propagating from the x-ray source ${\varvec{s}}$ to a detector pixel **d**. Along the ray $\overrightarrow{{\varvec{s}}{\varvec{d}}}$, we sequentially sample multiple coordinates denoted as ${{\varvec{t}}}_{i}={\varvec{s}}+{\alpha }_{i}({\varvec{d}}-{\varvec{s}})$, $0<{\alpha }_{i}<{\alpha }_{i+1}<1,\forall i$. The attenuation coefficients of these samples are queried with the forward pass of AFN and then discretely integrated; that is,

6 $$\widehat{{\varvec{p}}}\left({\varvec{d}}\right)={\sum }_{i}{h}_{\Theta }\left({{\varvec{t}}}_{i}\right)|{{\varvec{t}}}_{i+1}-{{\varvec{t}}}_{i}|,$$

where ${{\text{h}}}_{\Theta }(\cdot )$ denotes an AFN parameterized by $\Theta$. It should be noted that the projection estimation/rendering is model-dependent. Provided that a primary beam plus scatter model was used, AFN will be capable of scatter reduction when trained sufficiently. In this work, we stay within the primary beam-only model. The error between the estimated projection $\widehat{{\varvec{p}}}\left({\varvec{d}}\right)$ and the acquired projection ${\varvec{p}}({\varvec{d}})$ defined in Eq. (2) serves as the training loss to optimize the network’s representation:

7 $$\underset{\Theta }{{\text{argmin}}}{\sum }_{{\varvec{d}}}{\Vert \widehat{{\varvec{p}}}\left({\varvec{d}}\right)-{\varvec{p}}\left({\varvec{d}}\right)\Vert }^{2}, {\varvec{d}}\in {\mathbb{D}},$$

where ${\mathbb{D}}$ denotes the set of coordinates of all the acquired projection data. A trained AFN is intrinsically a continuous representation of the attenuation coefficients of the underlying object. The three-dimensional image reconstruction can be obtained by simply inputting the canonical coordinates of the imaging field. Alternatively, we may use AFN to emulate the cone-beam projection process described in Eq. (2) to obtain projection data with any imaging geometry. When the same imaging geometry (as that of the acquired data) is emulated, AFN can synthesize data that are unacquired. The unacquired synthesized projection data are spliced with the acquired projection data to yield complete projections, which can be subsequently reconstructed with any existing reconstruction methods.

### Clinical breast CT dataset

This study used de-identified projection datasets from 50 women assigned Breast Imaging-Reporting and Data System (BIRADS^30^) diagnostic assessment category 4 or 5. The clinical breast data were acquired under an institutional review board (IRB) approved (University of Arizona Human Subjects Protection Program, Protocol #1903470973) and Health Insurance Portability and Accountability Act (HIPPA) compliant research study (ClinicalTrials.gov Identifier: NCT01090687). All the research was performed in accordance with relevant guidelines/regulations. Informed consents were obtained from all participants. The projection data were acquired with a clinical prototype cone-beam breast CT scanner (KBCT 1000 prototype, Koning Corporation, West Henrietta, NY). The scanner employed a pulse-mode x-ray tube (RAD-71SP, Varex Imaging, Salt Lake City, UT) with 49 kVp and a *non-offset* flat panel detector (PaxScan 4030CB, Varian Medical Systems, Salt Lake City, UT) of size 40 cm × 30 cm. The detector was operated in 2 × 2 binning mode, resulting in 1024 × 768 pixels with a pixel pitch of 0.388 mm. The patient lies prone with one breast suspended through a tabletop opening into the imaging field (Fig. 1a). The x-ray tube and the detector were rotated about the breast to acquire 300 projection views uniformly across [0, $2\pi$). The scan time was approximately 10 s. The distance from the x-ray source to the axis of rotation (AOR) was 65 cm, and the distance from the source to the detector was 89.8 cm.

### Experimental setup

To emulate datasets acquired in a short scan in conjunction with an offset detector, we selected 225 out of 300 views covering 270 degrees where each projection view was truncated 256 out of 1024 pixels on the left. Note the 270° arc is symmetric about the head-feet direction and open towards the head (Fig. 1b). The truncated projection view is equivalent to the data acquired using a 30 × 30 cm^2^ detector with a *5 cm lateral shift*. The incomplete data result in an undersampling rate of $\frac{3}{4}\times \frac{3}{4}=\frac{9}{16}$. The full-scan data prior to truncation of the projections were reconstructed using the FDK algorithm at a 0.273 mm voxel size and served as the reference. Our AFN was trained on incomplete breast data and used to obtain three-dimensional image reconstructions or synthesize the missing projections during inference. We spliced AFN synthesized projections and the acquired incomplete data to yield complete data, which were subsequently reconstructed by FDK or FDK with the offset-detector weight^11^, referred to as FDK-M. For the spliced data, it is worth noting that the offset-detector weight was applied in a post-convolution step.

Our AFN approach was compared with three FDK methods using the (modified) Parker weight^23^, the offset-detector weight, or both weights. Specifically, the incomplete data were zero-filled to yield the same dimension as the complete data, and the weights were applied in a pre-convolution step as elemental-wise multiplications. In addition, the compressed sensing-based iterative reconstruction (FRIST^24^) was included for comparison. FRIST is known to suppress artifacts in the periphery and is initialized using FDK reconstructions. We thus performed two FRIST reconstructions using either of the two weighted FDK images as an initialization. We also trained two fully supervised networks independently to tackle the incomplete data problem, where the network inputs were obtained using either FDK with Parker weight or FDK with the offset-detector weight. We adopted a multi-slice residual dense network (MS-RDN)^26^ as the architecture, which was designed for breast CT reconstruction.

Our AFN was quantitatively evaluated using noise variance estimated in the adipose region, the signal difference (between adipose and fibroglandular tissues) to noise ratio (SDNR), and the full width at half maximum (FWHM) of calcifications in the mediolateral direction and the superior-inferior direction, respectively^13^. Statistical analysis was performed between our AFN and the reference FDK. A p-value less than 0.05 was considered to be statistically significant. For each image quality metric, we tested for normal distribution (Shapiro–Wilk’s test). If the normality assumption was satisfied, a paired t-test was performed to find statistical differences between our AFN method and the reference FDK method. Otherwise, a non-parametric Wilcoxon-signed rank test was used.

To examine the data sufficiency of AFN, we trained AFNs on a large-size breast data for different undersampling rates. Specifically, we laterally shifted the detector by 2.5 cm, 5 cm, and 7.5 cm and varied the view angle among 204°, 220°, and 270°, resulting in 9 combinations. Note that the combination of a 5 cm detector offset and 270° angular coverage corresponds to the emulated incomplete data acquisition geometry. The minimum angular coverage is 204° (= 180$^\circ +24^\circ$ fan angle) for this cone-beam geometry.

### Implementation

Our AFN consists of three fully-connected layers with a feature dimension of 64. Prior to the first fully-connected layer, we employed the hash grid encoding^22^ to accelerate training as well as to enhance the learning of high-frequency features. We used the default hash encoding parameters other than the hash table size. Since our breast CT problem in size (${2}^{29}$–${2}^{30}$ voxels) is similar to the gigapixel (in the scale of ${2}^{30}$ pixels) image representation task in the hash encoding paper^22^, we selected the same hash table size of ${2}^{23}$. At the end of the last fully-connected layer, we appended a custom activation function to enforce the non-negative constraint of attenuation coefficients. The activation function is the exponential function with its gradient clipped within $[-15, 15]$ to prevent vanishing or exploding gradients. ReLU activations were used for other layers.

An AFN was independently trained for each breast data. ADAM optimizer^31^ (${\beta }_{1}=0.9$, ${\beta }_{2}=0.99$, $\varepsilon =1\times{10}^{-15}$) was used with a weight decay regularization of $1\times{10}^{-6}$. The weight decay is the L2 norm on the network weights to penalize large weights. The learning rate was initialized at $1\times{10}^{-3}$ and decayed by one-third every 50 epochs for 250 epochs in total. The AFN training is designed to learn from all the acquired projection pixels ${\varvec{p}}\left(\beta ,u,v\right)$ at least once. To help AFN quickly glimpse the underlying imaging object, we let AFN *sparsely* iterate through, in an epoch, all the acquired projection views, i.e., ${{\varvec{p}}}_{\beta }\left(u,v\right)$. Thus, the number of batches in an epoch equals the number of acquired projection views. We selected a batch size of 2,048, which amounts to 2,048 randomly sampled pixels of a projection view. Note that the random sampling is non-repeating across epochs. The data training scheme that prioritizes iterating through all the acquired projection views helps AFN converge quickly within the first few epochs. A detector pixel ${\varvec{d}}$ together with the x-ray source ${\varvec{s}}$ can form a ray $\overrightarrow{{\varvec{s}}{\varvec{d}}}$, where we applied the stratified sampling approach^32^. The ray $\overrightarrow{{\varvec{s}}{\varvec{d}}}$ was first truncated within the imaging FOV and partitioned into 512 evenly spaced bins, within which one sample was uniformly drawn from. That many numbers of samples were selected mainly based on the reconstruction voxel pitch. Since coordinates are the inputs to the network, the effective batch size of our network equals 2048 × 512 = 1024 Ki, or roughly a million. This enormous batch size amounts to about 20 GB GPU memory, i.e., 20 KB per coordinate, yet greatly accelerates the network training as well as the network convergence^22^. The training of our AFN network took about two hours, and the rendering of 300 projection views took about 25 min on an NVIDIA RTX A6000 graphics card.

The MS-RDN^26^ consists of a high-resolution branch and a low-resolution branch. In each branch, we used four dense compression units (DCU), where each DCU is composed of eight modified dense blocks. The same architecture was used for network inputs obtained using FDK reconstruction with Parker weight or offset-detector weight. FDK reconstructions using complete projection data were used as network targets. The network was trained using L2 loss and was optimized using ADAM (${\beta }_{1}=0.9$, ${\beta }_{2}=0.99$, $\epsilon =1\times{10}^{-15}$) with a weight decay regularization of $1\times{10}^{-6}$. The learning rate was initialized at $1\times{10}^{-4}$ and decayed by one-third every 33 epochs for 100 epochs. Breast data from 20 subjects, 1 subject, and the remaining 29 subjects were used for training, validation, and test, respectively. With a batch size of four 256-by-256 patches randomly extracted from the coronal, sagittal, and axial planes, the training on an A6000 graphics card took about 22 h and 23.8 GB GPU memory.

The hyperparameters of FRIST except for the total variation (TV) regularization parameter $\alpha$ were fixed: $\beta =1$, ${\beta }_{{\text{redution}}} =0.995$, ${\alpha }_{{\text{reduction}}}=0.95$, ${\gamma }_{{\text{max}}}=0.95$, 100 total iterations, and 10 TV inner iterations. The TV regularization parameter $\alpha$ was finetuned for the incomplete data reconstruction problem and was set to 0.001. The FRIST algorithm took about 20–30 min for a breast dataset depending on the breast size.

AFN, MS-RDN, and an in-house FDK algorithm were implemented in PyTorch^33^. The FRIST algorithm was implemented using the TIGRE toolbox^34^, which supports forward and backward projections on GPU. The statistical analysis was performed in MATLAB (The MathWorks Inc., Natick, Massachusetts).

## Data Availability

The datasets generated during and/or analyzed during the current study are not publicly available due to patient privacy but are available from the corresponding author on reasonable request and institutional review.
